# SARS-CoV-2 infection predicts larger infarct volume in patients with acute ischemic stroke

**DOI:** 10.3389/fcvm.2022.1097229

**Published:** 2023-01-10

**Authors:** Manuela De Michele, Svetlana Lorenzano, Paola Piscopo, Roberto Rivabene, Alessio Crestini, Antonio Chistolini, Lucia Stefanini, Fabio M. Pulcinelli, Irene Berto, Roberta Campagna, Paolo Amisano, Marta Iacobucci, Carlo Cirelli, Anne Falcou, Ettore Nicolini, Oscar G. Schiavo, Danilo Toni

**Affiliations:** ^1^Emergency Department, Stroke Unit, Sapienza University of Rome, Rome, Italy; ^2^Department of Human Neurosciences, Sapienza University of Rome, Rome, Italy; ^3^Department of Neuroscience, Istituto Superiore di Sanità, Rome, Italy; ^4^Hematology, Department of Translational and Precision Medicine, Sapienza University of Rome, Rome, Italy; ^5^Department of Translational and Precision Medicine, Sapienza University of Rome, Rome, Italy; ^6^Department of Experimental Medicine, Sapienza University of Rome, Rome, Italy; ^7^Department of Molecular Medicine, Laboratory of Virology, Sapienza University of Rome, Rome, Italy; ^8^Neuroradiology Unit, Department of Human Neurosciences, Sapienza University of Rome, Rome, Italy

**Keywords:** stroke, SARS-CoV-2, COVID-19, coagulation, thrombosis, platelet activation, endothelium activation

## Abstract

**Background and purpose:**

Acute ischemic stroke (AIS) is a fearful complication of Coronavirus Disease-2019 (COVID-19). Aims of this study were to compare clinical/radiological characteristics, endothelial and coagulation dysfunction between acute ischemic stroke (AIS) patients with and without COVID-19 and to investigate if and how the SARS-CoV-2 spike protein (SP) was implicated in triggering platelet activation.

**Methods:**

We enrolled AIS patients with COVID-19 within 12 h from onset and compared them with an age- and sex-matched cohort of AIS controls without COVID-19. Neuroimaging studies were performed within 24 h. Blood samples were collected in a subset of 10 patients.

**Results:**

Of 39 AIS patients, 22 had COVID-19 and 17 did not. Admission levels of Factor VIII and von Willebrand factor antigen were significantly higher in COVID-19 patients and positively correlated with the infarct volume. In multivariate linear regression analyses, COVID-19 was an independent predictor of infarct volume (B 20.318, Beta 0.576, 95%CI 6.077–34.559; *p* = 0.011). SP was found in serum of 2 of the 10 examined COVID-19 patients. Platelets from healthy donors showed a similar degree of procoagulant activation induced by COVID-19 and non-COVID-19 patients' sera. The anti-SP and anti-FcγRIIA blocking antibodies had no effect in modulating platelet activity in both groups.

**Conclusions:**

SARS-CoV-2 infection seems to play a major role in endothelium activation and infarct volume extension during AIS.

## Introduction

Hypercoagulation and microthrombosis represent the typical features of Coronavirus disease 2019 (COVID-19) caused by Severe Acute Respiratory Syndrome Coronavirus 2 (SARS-CoV-2) (1). The pathogenic mechanisms are not yet fully understood, but multiple pathways are involved, including endothelialitis, endothelium dysfunction due to a direct (1) or pericyte-mediated (2) virus infection, a hyper-inflammatory state (“cytokine storm”), neutrophil activation and neutrophil extracellular traps (NETs) formation that trigger thrombosis in a complex phenomenon known as “thromboinflammation” (1, 3, 4). SARS-CoV-2 infection is also associated with platelet hyperactivity with increased platelet-platelet and platelet-leukocyte interactions (5). Spike protein (SP) is the SARS-CoV-2 homotrimeric protein located on the envelop that mediates the entrance of the virus into the target cells, mainly *via* the angiotensin-converting enzyme 2 (ACE2). Although the presence of ACE2 on platelets is controversial, some authors have demonstrated that SARS-CoV-2 may interact directly with platelets *via* ACE2 or, in alternative, *via* other receptors (i.e., TMPRSS2) and that recombinant anti-human ACE2 protein and anti-SP monoclonal antibody could inhibit SARS-CoV-2 SP-induced platelet activation (6). SARS-CoV-2 can also be taken up by platelets through endocytosis, leading to platelet apoptosis (7), and soluble SP (sSP) *per se* has been shown to directly activate platelets supporting blood hypercoagulation (8).

We recently detected the SP, but not the nucleocapsid protein (NP) or the SARS-CoV-2 RNA, in thrombi retrieved from the middle cerebral artery (MCA) of three COVID-19 patients with large vessel occlusion related acute ischemic stroke (AIS) and in a thrombus retrieved from the coronary artery of a COVID-19 positive patient with acute myocardial infarction, supporting a potential role of sSP, independently of the whole virus, in clot formation (9).

Indeed, SARS-CoV-2 antigens have been found in the blood of severe COVID-19 patients and correlated with disease progression (10).

COVID-19 and Vaccine-induced immune thrombotic thrombocytopenia (VITT), the catastrophic syndrome characterized by extensive venous and arterial thrombosis 5 to 30 days after the administration of the first dose of DNA viral-vector vaccines against the SARS-CoV-2, share a similar dysregulated interplay between platelets, innate immune effectors and coagulation factors, which ultimately lead to thromboinflammation (11). However, the antibodies against platelet factor-4 (PF4) which are the hallmark of VITT, are only sporadically found in COVID-19 patients, and in most cases, they are functionally inactive (12). A role of SP in VITT- and COVID-19-related discoagulopathy has been advocated by our group and others (13, 14). Based on all this data, we aimed to analyze the clinical/radiological characteristics, endothelial dysfunction and coagulation biomarkers of a cohort of AIS patients affected by COVID-19 in comparison with a cohort of AIS patients without COVID-19 and to verify whether, similarly to VITT, SP could play a pathogenic role in platelet activation in AIS patients with COVID-19. Furthermore, we aimed to evaluate whether COVID-19 independently predicts stroke lesion volume.

## Methods

### Patients and controls

A consecutive sample of AIS patients (>18 years of age) with COVID-19, admitted to the emergency department of our teaching hospital within 12 h of symptom onset, from March 2020 to May 2021, was included in the study. Diagnosis of SARS-CoV-2 infection was made by COVID-19 real-time reverse transcriptase-polymerase chain reaction (rRT-PCR) test on nasopharyngeal swab. For each patient, we collected demographic data, pre-stroke mRS, past medical history, vascular risk factors, and pre-stroke medications. Pre-stroke functional ability and stroke severity were assessed by using the modified Rankin scale (mRS) and National Institutes of Health Stroke Scale (NIHSS), respectively. COVID-19 severity was assessed by the Sequential Organ Failure Assessment (SOFA) score with a score of >1 indicating a severe disease (15).

As controls, we included a cohort of AIS patients without COVID-19, mostly admitted during the pre-pandemic period, whose clinical/radiological data were previously recorded in a dedicated database and whose serum and plasma samples were stored at −80°C for research purposes after obtaining patient informed consent.

Eligible patients were treated with IV thrombolysis or mechanical thrombectomy according to the current national and international guidelines (16–18). In both cases and controls, brain computed tomography (CT)/CT-Angiography (CTA) were performed in the acute phase, while multimodal 1.5 Tesla Magnetic Resonance Imaging (MRI) was obtained, where applicable, within 24 h from symptom onset. Neuroimaging data (including type of occluded vessel, infarct side and volume, presence of hemorrhagic transformation, collateral status by the Menon regional leptomeningeal score on CTA (19), and vessel recanalization assessed by using the Thrombolysis in Cerebral Infarction [TICI] score for patients receiving mechanical thrombectomy) was analyzed by two independent expert neuroradiologists. In particular, infarct volume was calculated on the MRI T2-FLAIR images through the A^*^B^*^C/2 formula (20). When it was not possible to perform MRI, CT scan was used as an alternative. Regarding collaterals, Menon scores of 0–10, 11–16, or 17–20 indicated poor, moderate, or good collateral circulation, respectively. Chest CT scan was also performed in all COVID-19 patients to evaluate the presence of SARS-CoV-2-related pulmonary infection.

Trial of ORG 10172 in Acute Stroke Treatment (TOAST) criteria were used to categorize stroke etiology (21).

All study participants gave a written informed consent. The study was approved by the Ethical Committee of our Institution and was conducted according to the Declaration of Helsinki.

### Laboratory testing

Venous blood samples for routine examination were collected at admission. Due to organizational issues, it was possible to perform a supplementary battery of blood tests only in a subset of 10 non-consecutive AIS patients per group. In these patients we analyzed coagulation parameters and biomarkers of endothelium dysfunction as follows: coagulation factors VIII and XIII; total levels of von Willebrand factor antigen (vWF:Ag), and its capability to adhere to platelet glycoprotein complex GPIb-IX-V (VWF:RCo). Furthermore, we also measured the following serum antibodies to PF4/polyanion by using a commercial enzyme immunoassay (IgG/IgA/IgM, Immucor, Lifecodes, Waukesha, WI, cat. # X-HAT45); serum SP by ELISA (My BioSource, San Diego, CA, USA, cat.#MBS7608267); Nucleocapside protein (NP) in the COVID-19 patient group alone by CLEIA (Lumipulse SARS-CoV 2-Ag—CND W0105040619—Fujirebio, cat.# 260340).

Antibody titers were measured using the LIAISON^®^ SARS-CoV-2 Trimeric S-IgG kit (DiaSorin S.p.A., Saluggia, Italy, cat# 311510) an indirect chemiluminescence immunoassay (CLIA) technology for the detection of serum IgG antibodies directed against SARS-CoV-2 trimeric SP. The assay quantification range is between 4.81 and 2080 BAU/mL (Binding Antibody Units/ml), with a cut-off value of 33.8 BAU/ml.

### Funcional platelet assay

Washed platelets [from 3 healthy donors (HDs)] were obtained by serial centrifugation and combined to a final concentration of 5 × 10^8^ cells/ml. Sera obtained from stroke patients or HDs were inactivated at 56°C for 30 min. After cooling and a short spin, sera were treated or not with 4 μg/ml of an antibody directed against the SARS-CoV-2 SP S1 subunit (Invitrogen, clone P06DHuRb) for 15 min at 37°C and then incubated for 10 min with washed platelets, preincubated or not with 20 μg/ml of the FcγIIa receptor-blocking antibody (Boster Biological Technology, clone IV.3). The platelet adhesive and pro-coagulant capacity were assessed on a BD Accuri C6 flow cytometer, by measuring the binding of PAC1-FITC (BD Bioscience), that recognizes the active form of integrin αIIbβ3, and of Annexin V-PE (Sony Biotechnology) that binds phosphatidylserine (Supplementary Figure S1).

### Statistical analysis

We performed a descriptive analysis; continuous variables were expressed as mean (±SD) or median (interquartile range [IQR]) as appropriate, while dichotomous and categorical variables were reported as counts, and proportion were calculated by dividing the numbers of events by the total number of patients, excluding missing or unknown cases. Student's *t*-test or Mann-Whitney *U* test and χ^2^ or Fisher exact test were used as appropriate for the comparison of demographics, clinical, radiological, and laboratory characteristics between the two groups of AIS patients with and without COVID-19. Correlation coefficients (Spearman ρ) were derived to quantify the association between biomarkers and infarct volume. Multivariate linear regression models (including a clinical model and clinical/biomarkers model) were used to adjust for the effects of potential confounders and to evaluate whether the presence of COVID-19 was an independent predictor of stroke lesion volume. Variables with *p*-values < 0.05 at univariate analysis for infarct volume were included in the multivariate models as well as other variables potentially influencing infarct volume regardless their univariate *p*-value. Given the small sample of patients included in the study, multivariate analysis should be considered as exploratory with a potential risk of model overfitting and the results should be interpreted with caution and deemed as not conclusive.

Statistical significance level was set at *p* < 0.05 for all analyses. Statistical analysis was performed using SPSS statistical software (IBM Corp, SPSS Statistics for Windows, Version 25, Armonk, NY).

## Results

### Patient characteristics

A total of 39 AIS patients were enrolled in this study: 22 (female 5; mean ± SD age 68.4 ± 16.1 years) with and 17 (female 5; mean ± SD age 71.4 ± 14 years) without COVID-19. Clinical and demographic characteristics are summarized in Table 1. Vascular risk factors were similar between the two groups except for a significant higher prevalence of smoking and atrial fibrillation among patients without COVID-19 (52.9 vs. 4.5%; *p* = 0.001 and 52.9 vs. 18.2%; *p* = 0.022, respectively). Overall, the most frequently reported stroke risk factor was arterial hypertension with similar proportions in both COVID-19 and non-COVID-19 patients (*p* = 0.704).

**Table 1:** Demographics and clinical characteristics of the overall study AIS patient population and by COVID-19 diagnosis.

	**All patients (*n* = 39)**	**AIS with COVID-19 (*n* = 22)**	**AIS without COVID-19 (*n* = 17)**	***p***
**Demographics and pre-stroke clinical characteristics**				
Age, mean (SD)	69.7 (15.1)	68.4 (16.1)	71.4 (14.0)	0.522
Age categories,^*^ years (%)				0.899
−0–57	10 (25.6)	6 (27.3)	4 (23.5)	0.793
−58–70	10 (25.6)	5 (22.7)	5 (29.4)	0.640
−71–80	11 (28.2)	7 (31.8)	4 (23.5)	0.573
- >80	8 (20.5)	4 (18.2)	4 (23.5)	0.686
Sex (females) (%)	10 (25.6)	5 (22.7)	5 (29.4)	0.721
Pre-mRS (%)				0.085
−0	26 (66.7)	12 (54.5)	14 (82.4)	0.071
−1	6 (15.4)	3 (13.6)	3 (17.6)	0.734
−2	6 (15.4)	6 (27.3)	0	**0.021**
−3	0	0	0	
−4	1 (2.6)	1 (4.5)	0	0.379
−5	0	0	0	
Hypertension (%)	30 (76.9)	16 (72.7)	14 (82.4)	0.704
Diabetes mellitus (%)	13 (33.3)	10 (45.5)	3 (17.6)	0.068
Smoking current (%)	10 (25.6)	1 (4.5)	9 (52.9)	**0.001**
Dyslipidemia (%)	13 (33.3)	8 (36.4)	5 (29.4)	0.648
Ischemic cardiomyopathy (%)	8 (20.5)	4 (18.2)	4 (23.5)	0.709
Atrial fibrillation (%)	13 (33.3)	4 (18.2)	9 (52.9)	**0.022**
Previous stroke/TIA (%)	5 (12.8)	2 (9.1)	3 (17.6)	0.636
Anemia (%)	2 (5.1)	2 (9.1)	0	0.495
Hypothyroidism (%)	2 (5.1)	1 (4.5)	1 (5.9)	1.0
Carotid stenosis >50% (%)	12 (30.8)	6 (27.3)	6 (35.3)	0.590
Valvulopathy (%)	5 (12.8)	1 (4.5)	4 (23.5)	0.147
Neoplasm (%)	8 (20.5)	5 (22.7)	3 (17.6)	1.0
Antiplatelets (%)	15 (38.5)	7 (31.8)	8 (47.1)	0.322
Anticoagulants (%)	6 (15.4)	3 (13.6)	3 (17.6)	1.0
Beta-blockers (%)	14 (35.9)	8 (36.4)	6 (35.3)	0.945
ACE-I/ACEIIR-I (%)	20 (51.3)	9 (40.9)	11 (64.7)	0.140
Diuretics (%)	9 (23.1)	4 (18.2)	5 (29.4)	0.465
Calcium antagonists (%)	5 (12.8)	3 (13.6)	2 (11.8)	1.0
Lipid lowering medications (%)	11 (28.2)	7 (27.3)	5 (29.4)	1.0
Blood glucose lowering medications (%)	7 (17.9)	5 (22.7)	2 (11.8)	0.438
**Stroke clinical and radiological characteristics**				
Stroke on awakening/unknown time of onset	17 (43.6)	14 (63.6)	3 (17.6)	**0.004**
Infarct location (%)				0.411
- Right	14/38 (36.8)	7/21 (33.3)	7 (41.2)	
- Left	22/38 (57.9)	12/21 (57.1)	10 (58.8)	
- Bilateral	0	0	0	
- Subtentorial	2/38 (5.3)	2/21 (9.5)	0	
Occlusion site (%)				0.063
- Top of ICA	1/38 (2.6)	1/21 (4.8)	0	0.368
- Tandem occlusion	4/38 (10.5)	2/21 (9.5)	2 (11.8)	0.825
- MCA-M1	19/38 (50.0)	6/21 (28.6)	13 (76.5)	**0.003**
- MCA-M2	6/38 (15.8)	4/21 (19.0)	2 (11.8)	0.546
- MCA-M3-M4	2/38 (5.3)	2/21 (9.5)	0	0.197
- Posterior circulation	1/38 (2.6)	1/21 (4.8)	0	0.368
- No occlusion	5/38 (13.2)	5/21 (23.8)	0	**0.033**
NIHSS at baseline, median (IQR)	15 (5–21)	11.50 (3.50–21.25)	16 (10.50–20.50)	0.263
NIHSS at 24 h, median (IQR)	6 (4–16.50)	6 (1.75–16.75)	6 (4–16.75)	0.822
IV thrombolysis (%)	12 (30.8)	1 (4.5)	11 (64.7)	**< 0.001**
Mechanical thrombectomy (%)	16 (41.0)	5 (22.7)	11 (64.7)	**0.008**
Time from stroke onset to IVT (min)		(*n* = 1)		0.343
- Mean (SD)	180.7 (104.5)	215	177.3 (109.5)	
- Median (IQR)	151 (120–215)	–	150.5 (115, 201.25)	
Time from stroke onset to groin puncture (min)		(*n* = 3)		**0.036**
- Mean (SD)	362.7 (212)	555 (120.13)	310.3 (203.5)	
- Median (IQR)	268 (208.8–463.3)	581 (424–n/a)	265.0 (205.0–308.0)	
Time from onset to recanalization (min)		(*n* = 2)		0.086
- Mean (SD)	412.1 (231.8)	589 (42.4)	376.7 (239.0)	
- Median (IQR)	307.5 (282.25–515.25)	589	298 (275–376.50)	
Hemorrhagic transformation (%)	9 (23.1)	3 (13.6)	6 (35.3)	0.142
Collateral circulation (menon score) (%)				**0.046**
- Good (17–20)	6/16 (37.5)	0	6/13 (46.2)	0.150
- Moderate (11–16)	6/16 (37.5)	3/3 (100)	3/13 (23.1)	**0.016**
- Poor (0–10)	4/16 (25.0)	0	4/13 (30.8)	0.283
Collateral circulation (Menon score) (%)				0.529
- Good/moderate	12 (75.0)	3/3 (100)	9/13 (69.2)	
- Poor (10–0)	4/16 (25.0)	0	4/13 (30.8)	
TICI				0.427
−2a	2/18 (11.1)	1/5 (20.0)	1/13 (7.7)	
−2b	3/18 (16.7)	0	3/13 (23.1)	
−3	13/18 (72.2)	4/5 (80.0)	9/13 (69.2)	
Infarct volume, cm^3^				
- Mean (SD)	52.49 (82.76)	75.32 (100.54)	23.96 (40.18)	0.063^†^
- Median (IQR)	14.70 (3.63–60.93)	25.95 (6.80–136.88)	9.0 (3.08, 25.80)	0.123^‡^
Secondary prevention therapy (%)				0.188
- Aspirin	16/24 (66.7)	11/13 (84.6)	5/11 (45.5)	
- Clopidogrel	3/24 (12.5)	2/13 (15.4)	1/11 (9.1)	
- DAPT	2/24 (8.3)	0	2/11 (18.2)	
- Warfarin	1/24 (4.2)	0	1/11 (9.1)	
- DOAC	1/24 (4.2)	0	1/11 (9.1)	
- Enoxaparin	1/24 (4.2)	0	1/11 (9.1)	
TOAST (%)				**0.040**
- LVO	8/39 (20.5)	4 (18.2)	4 (23.5)	0.686
- Cardioembolic	14/39 (35.9)	5 (22.7)	9 (52.9)	0.054
- SVO	4/39 (10.3)	2 (9, 1)	2 (11.8)	0.788
- Cryptogenic	12/39 (30.8)	11 (50.0)	1 (5.9)	**0.004**
- Other determined causes	1/39 (2.6)	0	1 (5.9)	0.255

^*^The four age categories were identified by using following quartiles: 25°: 57; 50°: 70; 75°: 80 years (for all patients, median 70 [IQR 57-80]).

Values are presented as mean (±SD), median (interquartile range [IQR]), or number (%) as appropriate.

Student's t-test or Mann-Whitney U test and χ^2^ or Fisher exact test were used as appropriate.

^†^Student t-test; ^‡^Mann-Whitney U test.

ACE-I/ACEIIR-I, angiotensin-converting enzyme-I inhibitors/angiotensin-converting enzyme-II receptor inhibitors; DAPT, double antiplatelet therapy; DOAC, direct oral anticoagulant; HU, Hounsfield unit; ICA, internal carotid artery; IQR, interquartile range; IV, intravenous; LVO, large vessel occlusion; MCA, middle cerebral artery; mRS, modified Rankin Scale; NIHSS, National Institutes of Health Stroke Scale; SD, standard deviation; SVO, small vessel occlusion; TIA, transient ischemic attack; TICI, thrombolysis in cerebral infarction; TOAST, Trial of Org 10172 in Acute Stroke Treatment. Bold values correspond to statistically significant results.

COVID-19 patients had stroke on awakening or of unknown time of onset significantly more frequently than non-COVID-19 patients (63.6 [14/22] vs. 17.6% [3/17], *p* = 0.004). The baseline median (IQR) NIHSS score was 15 [5–21.0]) with similar degree in both groups (11.50 [3.50–21.25] AIS with COVID-19 vs. 16 [10.50–20.50] AIS non-COVID-19, *p* = 0.263).

According to the TOAST classification, cardioembolic stroke tended to be more prevalent in non-COVID-19 compared to COVID-19 patients (52.9 [9/17] vs. 22.7% [5/22], *p* = 0.054); conversely, the latter had more frequently a cryptogenic stroke (50.0 [11/22] vs. 5.9% [1/17], *p* = 0.004) (Table 1).

Patients with COVID-19 received less frequently IV tissue-type plasminogen activator (tPA) (4.5 [1/22] vs. 64.7% [11/17], *p* < 0.001) or mechanical thrombectomy (22.7 [5/22] vs. 64.7% [11/17], *p* = 0.008) with longer median time intervals from stroke onset to endovascular treatment (581 vs. 265 min., *p* = 0.036) (Table 1).

Among the COVID-19 AIS patients, the mean time from COVID-19 diagnosis and stroke onset was 4.6 days (±7.1 SD) (Supplementary Table S1). Typical ground glass pneumonia was detected in 17/22 (77.3%) COVID-19 patients, with 7/17 (41.2%) exhibiting more than 50% of lung parenchyma involvement. None of the COVID-19 patients met the sepsis criteria according to the Sepsis-3 International Consensus (15), while 8/19 (42.1%) of them had a SOFA score ≥ 1.

Only one patient had received the first dose vaccination against SARS-CoV-2 (Pfizer BioNTech) 10 days before stroke onset. The patient had also a pneumonia and SOFA score of 2.

Demographics, clinical characteristics, and laboratory data of a subset of 10 non-consecutive AIS patients with COVID-19 for whom additional specific biomarkers were measured, were overall similar to those of the remaining 12 COVID-19 patients except for a higher COVID-19 severity (SOFA score > 1: 75 [6/8] vs. 18.2% [2/11], *p* = 0.024; mean [SD] SOFA score: 2.2 [1.4] vs. 0.82 [1.0], respectively) (Supplementary Table S2).

### Routine laboratory testing

Concerning the routine laboratory tests at admission, in the COVID-19 cohort we observed significantly lower lymphocyte count compared with the non-COVID-19 counterpart (mean [SD] 1.35 [0.92] × 10^3^/μL vs. 2.30 [1.25] × 10^3^/μL; *p* = 0.035) and significantly higher levels of LDH (461.56 [404.06] vs. 219.57 [47.18] mg/dL; *p* = 0.034) (Supplementary Table S3).

### Coagulation profile, markers of endothelium dysfunction

**Figure 1:**
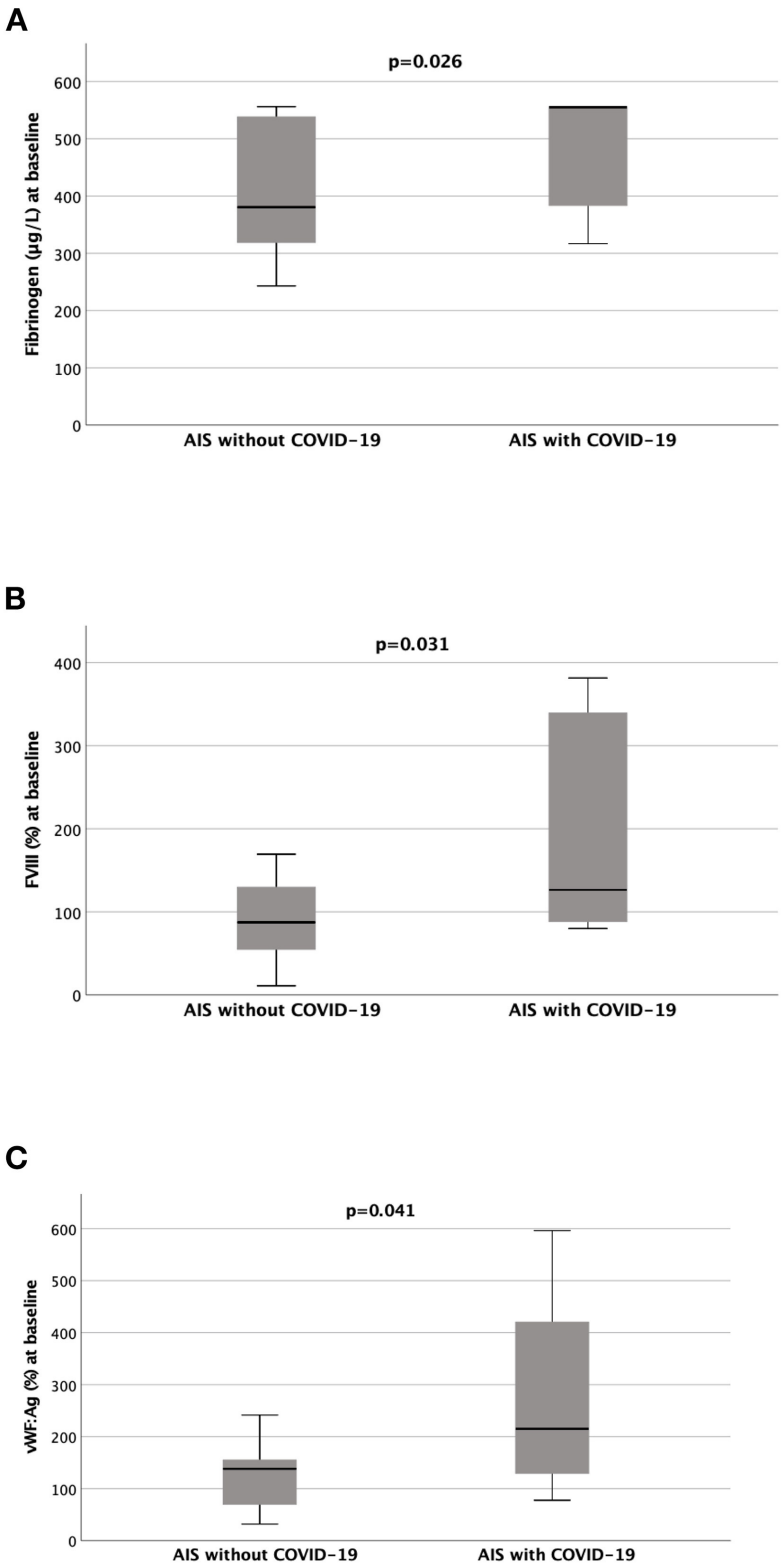
Fibrinogen, FVIII and VWF:Ag circulating levels are significantly higher in COVID-19 acute ischemic stroke patients than in the non-COVID-19 acute ischemic stroke patients. The graphs show the differences (Mann-Whitney *U* test) in coagulation parameters between acute ischemic stroke (AIS) patients with COVID-19 and without COVID-19: **(A)** Fibrinogen (without COVID-19, *n* = 14 vs. with COVID-19, *n* = 20); **(B)** Factor VIII (FVIII) (without COVID-19, *n* = 10 vs. with COVID-19, *n* = 8); **(C)** von Willebrand Factor Antigen (vWF:Ag) (without COVID-19, *n* = 9 vs. with COVID-19, *n* = 8).

As regards the coagulation profile, fibrinogen levels were significantly higher in COVID-19 patients than in the non-COVID-19 patients (mean [SD], 486.25 [92.69] vs. 405.21 [108.83] mg/dL; *p* = 0.026) (Supplementary Table S3 and Figure 1A). Based on the available data, D-Dimer levels were elevated at admission in the majority of both COVID-19 and non-COVID-19 patients, however, they were not significantly different between the two groups (*p* = 0.150) (Supplementary Table S3). INR and aPTT were normal.

In a subset of 10 AIS patients per group, coagulation Factor VIII was increased in cases compared to controls (196.18 [132.84] vs. 87.89 [52.79]%; *p* = 0.031) (Supplementary Table S3 and Figure 1B) whereas Factor XIII was within the normal range in both patient groups. Overall, we observed increased circulating levels of VWF:Ag and VWF:RCo, which were however higher in the COVID-19 group than in the non-COVID-19 group with a statistically significant difference reached for the VWF:Ag levels (mean [SD], 275.39 [184.97]% *p* = 0.041) (Supplementary Table S3 and Figure 1C).

Admission levels of Factor VIII and vWF:Ag positively and significantly correlated with infarct volume in the overall study population (Spearman's rho = 0.539, *p* = 0.026 and 0.697, *p* = 0.003, respectively) (Supplementary Figure S2), as well as in the two COVID-19 and non-COVID-19 patients although without reaching statistical significance.

### Spike protein (SP), nucleocapside protein, antibodies against SARS-CoV-2, antibodies anti-platelet factor-4 (PF-4)

Only 2 out of the 10 AIS/COVID patients with available additional biomarkers showed a measurable level of SP in the serum. Overall, no substantial differences in demographics, clinical and laboratory data were observed between SP-positive and SP-negative patients (Supplementary Table S4).

We could not detect NP in the serum of both cases and controls.

Among the 4 AIS with COVID-19 screened for the IgG against SARS-CoV-2 SP level, only 1 patient, who received 10 days earlier, the first dose of Pfizer BioNTech vaccine against COVID-19, showed high antibody titer (Supplementary Table S1).

Patients' serum of both groups did not show detectable levels of pan antibodies (IgG, IgM and IgA) to PF4-polyanion complexes.

### Platelet activation assay

**Figure 2:**
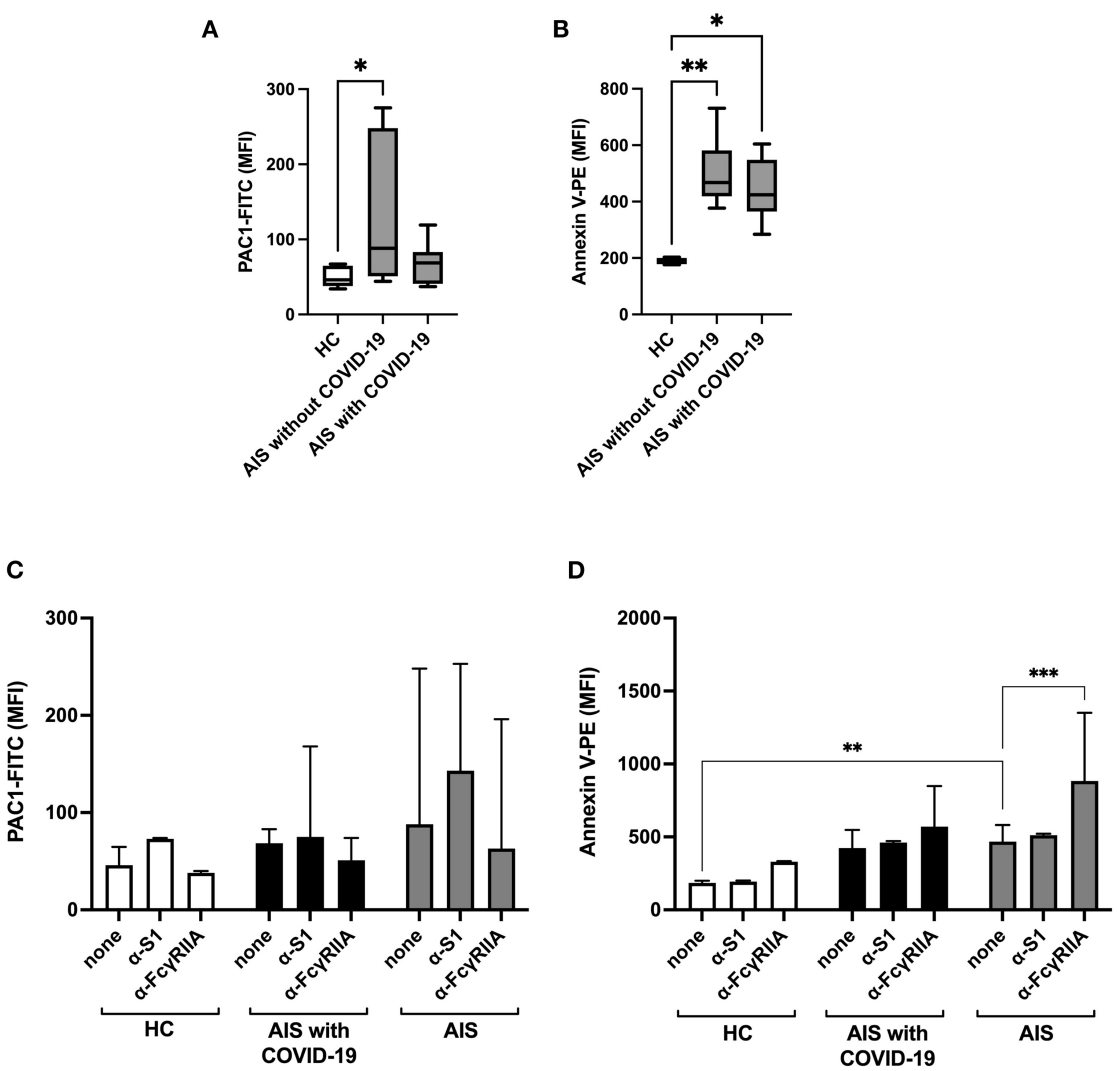
Sera from AIS patients, with or without COVID-19, induce activation of platelets from healthy controls. Flow cytometric analysis of **(A, C)** integrin activation (shown as median fluorescence intensity, MFI, of PAC1-FITC) and of **(B, D)** phosphatidylserine exposure, i.e., platelet pro-coagulant activity (shown as MFI of Annexin V-PE) of washed healthy platelets (of *n* = 3 healthy donors combined) incubated with sera from acute ischemic stroke (AIS, *n* = 10) patients, with or without COVID-19, or of healthy controls (HC, *n* = 6), in the presence or absence of blocking antibodies for the spike protein subunit 1 protein (α-S1) or the FcγRIIA (α-FcγRIIA) [Kruskal Wallis test for **(A, B)**; two-way ANOVA for **(C, D)**]. ^*^p < 0.05, ^**^p < 0.01, ^***^p < 0.001.

Plasma from AIS patients without COVID-19 induced a significant increase in the basal activation of integrin αIIbβ3 in the platelets of HCs, while plasma from AIS patients with COVID-19 had a modest non-significant effect (Figure 2A). Conversely, plasma from stroke patients either with or without COVID-19 induced a significant increase of phosphatidylserine exposure, which is indicative of a pro-coagulant activity (Figure 2B). Pre-incubation with a blocking antibody for the S1 protein or inhibition of the FcγRIIA, the platelet receptor for immune complexes, did not interfere with the upregulation of the platelet function (Figures 2C, D).

### Neuroimaging findings

Neuroimaging data are included in Table 1. As regards the infarct volume, we found a borderline statistically significant between-group difference with a median (IQR) infarct volume of 25.95 cm^3^ (6.80–136.88) in the COVID-19 patients and of 9.0 cm^3^ (3.08, 25.80) in the non-COVID-19 counterpart (*p* = 0.123) (Supplementary Figure S3).

Lepto-meningeal collaterals significantly differ between the two groups (*p* = 0.046) with COVID-19 patients having moderate collateral circulation (Menon score 11–16: 3/3, 100% vs. 3/13, 23.1%; *p* = 0.016) and non-COVID-19 patients having a larger variety of Menon scores from good (Menon score 17–20: 6/13, 46.2%) to poor (Menon score 0–10: 4/13, 30.8%) collateral status. There were no statistically significant between-group differences in terms of recanalization rate. Hemorrhagic transformation of the index infarct was found in 3/22 (13.6%) COVID-19 patients and in 6/17 (35.3) AIS patients without COVID-19 (*p* = 0.142).

At univariate analyses infarct volume resulted associated with the diagnosis of COVID-19 with a trend toward the statistical significance (*p* = 0.123) and significantly associated with the presence of internal carotid artery stenosis >50% (*p* = 0.009) and a clinical history of valvulopathy (*p* = 0.035). Furthermore, infarct volume correlated with baseline NIHSS (Spearman's rho = 0.579, *p* < 0.001), admission levels of endothelium dysfunction biomarkers (FVIII: Spearman's rho = 0.539, *p* = 0.026; vWF:Ag: Spearman's rho = 0.697, *p* = 0.003; vWF:RCo: Spearman's rho = 0.547, *p* = 0.023) and platelet count (Spearman's rho = 0.511, *p* = 0.002).

In multivariate linear regression analyses, COVID-19 resulted as an independent predictor of infarct volume both in the clinical (B 20.318, Beta 0.576, 95% CI 6.077–34.559; *p* = 0.011) and in the clinical/biomarker (B 35.440, Beta 0.837, 95% CI 3.320–67.560; *p* = 0.038) models (Table 2).

**Table 2:** Multivariate analysis for infarct volume.

	**Unstandardized B**	**Standardized coefficient beta**	**95% CI for B**	***p***
**Model 1**			
COVID-19	31.641	0.675	15.538.47.745	0.002
Baseline NIHSS	0.915	0.479	0.258–1.572	0.013
**Model 2**			
COVID-19	35.440	0.837	3.320–67.560	0.038

Model 1 (Clinical): adjusted for diagnosis of COVID-19, baseline NIHSS, IV thrombolysis, mechanical thrombectomy, onset-to-groin puncture time, onset-to-recanalization time, collateral status, TICI, ICA stenosis >50%, valvulopathy.

Model 2 (Clinical/Biomarkers): adjusted for variables included in Model 1 and for baseline FVIII, vWF:Ag, vWF:RCo, and platelet count.

ICA, internal carotid artery; IV, intravenous; NIHSS, National Institutes of Health Stroke Scale; TICI, thrombolysis in cerebral infarction.

## Discussion

The relative risk for the occurrence of AIS during COVID-19 is not very high ranging between 0.5 and 1.3%. However, since this risk is 3- to 4-fold greater compared to that of non-infected hospitalized historical or contemporary control cohorts (11) and since the outcome of AIS patients with COVID-19 appear worse both in terms of initial stroke severity and functional outcome/mortality (11) this subgroup of infected patients deserves a particular attention. In keeping with a previous work (22), in our study we showed that endothelium dysfunction could play a fundamental role in the pathogenesis of SARS-CoV-2-related cerebral arterial thrombosis. Additionally, taking into account that the multivariate analysis is exploratory in nature due to the small number of patients included in the study, we found that COVID-19 seems to predict the infarct volume extension, regardless the baseline stroke severity, the reperfusion treatments, the onset-to-groin puncture and onset-to-recanalization times, the collateral circulation status, the achieved recanalization degree or the biomarkers of endothelium dysfunction. Moreover, our data reveals that an unspecified factor or more factors from serum of AIS patients, other than the virus and immune complexes, promote platelet activation, in particular the propensity of platelets to become pro-coagulant. Finally, this study confirmed that SARS-CoV-2 sSP can be found in the sera of AIS patients with COVID-19. Taken together our data suggest that in a subset of AIS patients with moderate COVID-19, sSP in cooperation with the systemic inflammatory state, may induce a pro-inflammatory and pro-thrombotic endothelium phenotype with thrombosis extension and development of larger infarct volume despite a prompt recanalization of the occluded vessel.

vWF, an adhesive glycoprotein synthesized by endothelial cells and megakaryocytes, and coagulation factor VIII seems to be the main players in AIS COVID-19 clot formation in our study. Primary functions of vWF are facilitation of platelet adhesion to subendothelium when exposed to vascular injury and cooperation in clot growth and stabilization, in concert with fibrinogen, and in platelet–platelet interactions. In addition, vWF acts as a carrier protein for FVIII in plasma, protecting it from proteolytic degradation (23). SARS-CoV-2 or SP *per se* can directly or through a pericyte-mediated mechanism (2), activate endothelium cells or endothelialitis can be secondary to systemic inflammation (2, 3). In this regard, we have found a marked increase of fibrinogen levels in the COVID-19 patient group compared to AIS patients without COVID-19, whereas D-dimer levels were similar in both groups. In comparison to VITT, in which the trigger of platelet activation may be the abnormal vaccine-induced sSP (13) and the maladaptive immunity plays a fundamental role in pathogenesis (24), and in comparison to critically ill COVID-19 patients, where platelets are significantly altered to a more active phenotype and this activation is modulated by immune complexes (25), in COVID-19 related-AIS, sSP and immune complexes seems to have a secondary role. In fact, although we did not have the opportunity to study *in vivo* the platelets of the AIS patient of our cohort, in our experiments we observed a similar pattern of HDs' platelet activation after incubation with sera from AIS with and without COVID-19 patients and, moreover, neither SP antibody nor anti-FcγRIIa monoclonal antibody inhibited this activation suggesting a minor role of sSP and immune-mediated mechanisms in this subset of patients. Interestingly, serum of both groups of AIS patients stimulated a procoagulant phenotype of platelet activation. An enhanced procoagulant activity of blood cells has been demonstrated after AIS (26) and thromboinflammation is a well-recognized phenomenon in stroke (27). Our data can indicate that during the course of COVID-19, in moderately ill patients and at least in the acute phase of stroke, platelet activation does not seem to be driven by the SARS-CoV-2 infection.

We found detectable serum levels of SP but not of NP in 2 out of 10 screened patients, suggesting that in these cases only the antigen without the whole virus was in the blood stream. This data is in line with those recently published by an Italian group in which circulating SP has been found in 30.4% of severely ill COVID-19 patients (28). The authors have also demonstrated *in vitro* the ability of S1 to promote the release of vWF in the luminal surface of endothelium, leading to platelet adhesion and aggregation and consequent propagation of the prothrombotic effect on endothelium. This peculiar mechanism, which might contribute to the occurrence of the so called “no-reflow phenomenon” (29), could explain the reason why in our study COVID-19 AIS patients experienced larger infarct volume despite an effective recanalization therapy. SP is not easily detectable in blood of COVID-19 patients according to data from Ogata et al. (10). These authors found SP only in 5 of the 64 COVID-19 positive patients included in their study while the S1 subunit, which harbors the receptor-binding domain (30), was detected in 41 patients. We agree with the hypothesis formulated by the authors who suppose that free SP antigen in plasma could be proteolytically cleaved, releasing the S1 subunit, and the remaining fragment could be undetectable by the used assay. Another hypothesis is that SP viral antigen could be cleared up from plasma by the anti-SARS-CoV-2 immune response.

Interestingly, we recently described SP in a retrieved thrombus from MCA of one of the two AIS patients with COVID-19 having detectable serum SP level (9).

The study has limitations including the small sample of patients, the lack of an *in vivo* study of platelet activity, of dosage of the soluble S1 subunit in addition to sSP and of the examination of neutrophil extracellular traps (NETs), which play a role in both AIS and COVID-19 pathogenesis.

In conclusion, we can suppose that in addition to asymptomatic or mild self-limiting infection and critical illness with severe airway inflammation that can lead to acute respiratory distress syndrome, usually associated with a fatal outcome, there is, in the middle, a condition characterized by non-severe pulmonary infection. Nevertheless, these moderately ill patients can experience arterial thrombotic complications, probably not primarily linked to SARS-CoV-2- or SP-related platelet activation and to immune system derangements or aberrant glycosylation of anti-SARS-CoV-2 spike IgG immune complexes (25), but potentially associated to a direct effect of sSP or SARS-CoV-2 and inflammatory mediated mechanisms on endothelium. However, further larger studies are warranted to confirm our hypothesis.

## Data availability statement

The raw data supporting the conclusions of this article will be made available by the authors, without undue reservation.

## Ethics statement

The studies involving human participants were reviewed and approved by Comitato Etico del Policlinico Umberto I Hospital, Sapienza University of Rome. The patients/participants provided their written informed consent to participate in this study.

## Author contributions

MD conceived and designed the study, enrolled the patients, interpreted the results, and prepared the original manuscript. SL performed the statistical analysis, contributed to the manuscript draft preparation, edited the tables and the graphical abstract, and critically reviewed the manuscript. PP, RR, and ACr performed the biomarkers analysis, interpreted the data, and critically reviewed the manuscript. ACh performed the coagulation and endothelium markers analysis and critically reviewed the manuscript. LS conceived and performed the functional platelet assay, interpreted the results, edited Figure 2 and critically revised the manuscript. FP measured the serum antibodies to PF4/polyanion and revised the manuscript. IB and PA enrolled the patients, participated in data collection, interpreted the results and revised the manuscript. RC detected the serum IgG antibodies directed against SARS-CoV-2 trimeric Spike Protein and revised the manuscript. MI and CC evaluated and processed the neuroimaging data and revised the manuscript. AF, EN, and OS enrolled the patients and critically reviewed the manuscript. DT critically reviewed and edited the manuscript. All authors contributed to the article and approved the submitted version.
