# Supplementary material for: SARS-CoV-2 infection predicts larger infarct volume in patients with acute ischemic stroke
Source: Front Cardiovasc Med. 2023 Jan 10;9:1097229. doi: 10.3389/fcvm.2022.1097229 (PMC9871539; doi:10.3389/fcvm.2022.1097229)
Supplement: Supplementary file 1 [file Table_1.DOCX]

**SUPPLEMENTAL MATERIAL**

**COVID-19 and acute ischemic stroke: novel insight into pathophysiological mechanisms of thrombosis**

Manuela De Michele (MD, phD, MSc),^a^ Svetlana Lorenzano (MD, phD, MSc),^b^ Paola Piscopo (phD),^c^ Roberto Rivabene (phD),^c^ Alessio Crestini (phD),^c^ Antonio Chistolini (MD),^d^ Lucia Stefanini (phD),^e^ Fabio Pulcinelli (MD),^f^ Irene Berto (MD),^a^ Roberta Campagna,^g^ Paolo Amisano (MD),^b^ Marta Iacobucci (MD),^h^ Carlo Cirelli (MD),^h^ Anne Falcou (MD, phD),^a^ Ettore Nicolini (MD, phD),^a^ Oscar G. Schiavo (MD),^a^ Danilo Toni (MD)^b^

^a^ Emergency Department, Stroke Unit, Sapienza University of Rome, Rome, Italy

^b^ Department of Human Neurosciences, Sapienza University of Rome, Rome, Italy

^c^ Department of Neuroscience, Istituto Superiore di Sanità, Viale Regina Elena, 299; Rome. Italy

^d^ Hematology, Department of Translational and Precision Medicine, Sapienza University of Rome, Rome, Italy

^e^ Department of Translational and Precision Medicine, Sapienza University of Rome, Rome, Italy

^f^ Department of Experimental Medicine, Sapienza University of Rome, Rome, Italy

^g^Department of Molecular Medicine, Laboratory of Virology, Sapienza University of Rome, Rome, Italy

^h^ Neuroradiology Unit, Department of Human Neurosciences, Sapienza University of Rome, Rome, Italy

**Keywords:** stroke, SARS-CoV2, COVID-19, coagulation, thrombosis, platelet activation, endothelium activation

**Running title:** COVID-19 and stroke

Corresponding Author:

Manuela De Michele, MD, PhD, MSc

Emergency Department, Stroke Unit, Sapienza University of Rome

Viale del Policlinico, 155 – 00185 Rome - Italy

Phone number: 06/49979529

Fax number: 06/49979526

Email: M.DeMichele@policlinicoumberto1.it

**List of reference values of laboratory tests**

| **Laboratory test** | **Reference values** |
| --- | --- |
| Albumin g/dL | 3.5-5.2 |
| Antithrombin III (%) | 80 - 120 |
| aPTT, sec | 0.8-1.2 |
| C3 (mg/dL) | 90 - 180 |
| C4 (mg/dL) | 10 - 40 |
| CRP, mg/dL | 28-72 |
| D-dimer, μg/L | 0-550 |
| Factor VIII (%) | 58 - 130 |
| Factor XIII (%) | 64 - 140 |
| Fibrinogen, mg/dL | 200-400 |
| INR | 0.8-1.2 |
| LDH, UI/L | 135-225 |
| Lymphocytes, x10^3^/μL | (1-3.2) |
| Myoglobin, ng/ml | (28-72) |
| PLT, x10^3^/μL | 150-450 |
| VWF:Antigen (%) | 41-130 |
| VWF:RiCof (%) | 41-124 |

**Table S1. Characteristics of COVID-19 patients**

|  | **AIS with COVID-19**  **(n=22)** |
| --- | --- |
| Time from COVID-19 diagnosis and stroke (days)  - mean (SD)  - median (IQR) | 4.6 (7.1)  1 (0, 8.50) |
| Anti-SP antibodies (IgG), positive (%) | 1/4 (25.0) |
| Anti-SP antibodies (IgG), BAU/ml (range test 4.81-2080)  - mean (SD)  - median (IQR) | 390.20 (759.91)  13 (4.80-1152.80) |
| SP positive, % | 2/10 (20) |
| SP (pg/ml), mean (SD) | n=10  0.38 (0.80) |
| Anti-PF4 positive (ELISA; RVs: OD_405_ <0.5) | 0/10 |
| Chest CT (%) | 21 (95.5) |
| Pneumonia (%)  - <20% (mild)  - 20-50% (moderate)  - >50% (severe)  - Bilateral pleural effusion | 17 (77.3)  4/17 (23.5)  4/17 (23.5)  7/17 (41.2)  2/17 (11.8) |
| Sepsis (%) | 0 |
| MOF (%) | 0 |
| SOFA (%)  - 0  - 1  - 2  - 3  - 4 | 6/20 (30.0)  6/20 (30.0)  3/20 (15.0)  3/20 (15.0)  2/20 (10.0) |
| SOFA  - media (SD)  - mediana (IQR) | 1.52 (1.37)  1 (0-3) |
| SOFA >1 (%) | 8/19 (42.1) |
| Hemogasanalysis, mean (SD)  - pH  - pO2  - pCO2  - Lactates  - SpO2  - P/F RATIO  - HCO3^-^ | 7.43 (0.04)  98.23 (44.93) (median [IQR]: 88.00 [74-97.50])  32.15 (7.56)  2.10 (1.07)  97.52 (1.67)  358.46 (99.62) (median [IQR]: 400 [272.50-433.50])  22.41 (4.35) |

Values are presented as mean (±SD), median (interquartile range [IQR]), or number (%) as appropriate.

Student’s t test or Mann-Whitney U test and χ2 or Fisher exact test were used as appropriate.

Abbreviations: Anti-PF-4, anti-platelet factor 4; RVs: reference values; OD: optical density; CT, computerized tomography; IgG, Immunoglobulin G; IQR, interquartile range; MOF, multiorgan failure; P/F ratio, PO2/FIO2 ratio; SD, standard deviation; SOFA, Sequential Organ Failure Assessment; SP, spike protein.

**Table S2. Comparison of demographics and clinical characteristics between the 10 AIS patients with COVID-19 undergoing to biomarkers measurements and the remaining COVID-19 patients**

|  | **AIS pts with COVID-19 with biomarker available**  **(n=10)** | **AIS pts with COVID-19 without biomarker available**  **(n=12)** | **p** |
| --- | --- | --- | --- |
| **Demographics and pre-stroke clinical characteristics** | | | |
| Age, mean (SD) | 72.8 (17.6) | 64.8 (14.5) | 0.252 |
| Sex (females) (%) | 3 (30) | 2 (16.7) | 0.624 |
| Pre-mRS (%)  - 0  - 1  - 2  - 3  - 4  - 5 | 6 (60)  1 (10)  2 (20)  0  1 (10)  0 | 6 (50.0)  2 (16.7)  4 (33.3)  0  0  0 | 0.608 |
| Hypertension (%) | 7 (70) | 9 (75.0) | 1.0 |
| Diabetes mellitus (%) | 5 (50) | 5 (41.7) | 1.0 |
| Smoking (current?) (%) | 0 | 1 (8.3) | 1.0 |
| Dyslipidemia (%) | 5 (50) | 3 (25.0) | 0.378 |
| Ischemic cardiomyopathy (%) | 3 (30) | 1 (8.3) | 0.293 |
| Atrial fibrillation (%) | 3 (30) | 1 (8.3) | 0.293 |
| Previous stroke/TIA (%) | 1 (10) | 1 (8.3) | 1.0 |
| Anemia (%) | 1 (10) | 1 (8.3) | 1.0 |
| Hypothyroidism (%) | 0 | 1 (8.3) | 1.0 |
| Carotid stenosis >50% (%) | 2 (20) | 4 (33.3) | 0.646 |
| Valvulopathy (%) | 0 | 1 (8.3) | 1.0 |
| Neoplasm (%) | 2 (20) | 3 (25.0) | 1.0 |
| Antiplatelets (%) | 5 (50) | 2 (16.7) | 0.172 |
| Anticoagulants (%) | 2 (20) | 1 (8.3) | 0.571 |
| Beta-blockers (%) | 5 (50) | 3 (25.0) | 0.378 |
| ACE-I/ACEIIR-I (%) | 6 (60) | 3 (25.0) | 0.192 |
| Diuretics (%) | 2 (20) | 2 (16,7) | 1.0 |
| Calcium antagonists (%) | 1 (10) | 2 (16,7) | 1.0 |
| Lipid lowering medications (%) | 4 (40) | 2 (16,7) | 0.348 |
| Blood glucose lowering medications (%) | 3 (30) | 2 (16,7) | 0.624 |
| **Stroke clinical and radiological characteristics** | | | |
| Stroke on awakening / unknown time of onset | 6 (60) | 8 (66.7) | 1.0 |
| Infarct location (%)  - Right  - Left  - Bilateral  - Subtentorial | 4 (40)  6 (60)  0  0 | 3/11 (27.3)  6/11 (54.5)  2/11 (18.2)  0 | 0.350 |
| Occlusion site (%)  - Top of ICA  - Tandem occlusion  - MCA-M1  - MCA-M2  - MCA-M3-M4  - Posterior circulation  - No occlusion | 1 (10)  0  4 (40)  3 (30)  0  0  2 (20) | 2/11 (18.2)  0  2/11 (18.2)  1/11 (9.1)  2/11 (18.2)  1/11 (9.1)  3/11 (27.3) | 0.250 |
| NIHSS at baseline, median (IQR) | 17 (4.75-23) | 6.5 (2-19.75) | 0.262 |
| NIHSS at 24 h, median (IQR) | 8 (4-19) | 6 (0-16.0) | 0.710 |
| IV thrombolysis (%) | 1 (10) | 0 | 0.455 |
| Mechanical thrombectomy (%) | 4 (40) | 1 (8.3) | 0.135 |
| Time from stroke onset to IVT (min)  - mean (SD)  - median (IQR) | (n=1)  215  - | - | - |
| Time from stroke onset to groin puncture (min)  - mean (SD)  - median (IQR) | (n=3)  555 (120.1)  581 (424-n/a) | - | - |
| Time from onset to recanalization (min)  - mean (SD)  - median (IQR) | (n=3)  589 (42.43)  - | - | - |
| Hemorrhagic transformation (%) | 3 (30) | 0 | 0.078 |
| Collateral circulation (Menon score) (%)  - good1(17-20)  - moderate (16-11)  - poor (10-0) | 0/2  2/2 (100)  0/2 | 0  1 (100.0)  0 | - |
| Collateral circulation (Menon score) (%)  - good/moderate  - poor (10-0) | 2/2 (100)  0/2 | 1 (100.0)  0 | - |
| TICI  - 2a  - 2b  - 3 | 1/4 (25)  0  3/4 (75) | 0  0  1/1 (100.0) | 1.0 |
| Infarct volume, cm^3^  - mean (SD)  - median (IQR) | 73.47 (108.25)  25.95 (7.80-105.35) | 77.17 (98.05)  36.05 (0.38-158.18) | 0.910 |
| Secondary prevention therapy (%)  - Aspirin  - Clopidogrel  - DAPT  - Warfarin  - DOAC  - Enoxaparin | 5/6 (83.3)  1/6 (16.7)  0  0  0  0 | 6 (85.7)  1 (8.3)  0  0  0  0 | 1.0 |
| TOAST (%)  - LVO  - Cardioembolic  - SVO  - Cryptogenic  - Other determined causes | 2 (20)  3 (30)  0  5 (50)  0 | 2 (16.7)  2 (16.7)  2 (16.7)  6 (50.0)  0 | 0.547 |
| **Characteristics of COVID-19** | | | |
| Time from COVID-19 diagnosis and stroke  (days)  - mean (SD)  - median (IQR) | 6.5 (8.7)  4 (0-12.25) | 3.6 (6.2)  1.0 | 0.426 |
| Anti-SP antibodies (IgG), positive (≥4.81) (%) | 1/4 (25) | 0 | - |
| Anti-SP antibodies (IgG), BAU/ml  (test range 4.81-2080)  - mean (SD)  - median (IQR) | (n=4)  390.20 (759.91)  13 (4.80-1152.80) | - | - |
| SP, pg/ml, mean (SD) | 0.38 (0.80) | - | - |
| Anti-PF4 positive (%) | 0 | 0 | - |
| Chest CT (%) | 9 (90) | 12 (100.0) | 0.455 |
| Pneumonia (%)  - <20% (mild)  - 20-50% (moderate)  - >50% (severe)  - Bilateral pleural effusion | 8 (80)  1/9 (11.1)  3/9 (33.3)  3/9 (33.3)  2/9 (22.2) | 8/12 (66.7)  3/8 (37.5)  1/8 (12.5)  4/8 (50.0)  0 | 0.646 |
| Sepsis (%) | 0 | 0 | - |
| MOF (%) | 0 | 0 | - |
| SOFA (%)  - 0  - 1  - 2  - 3  - 4 | 1/9 (11.1)  2/9 (22.2)  2/9 (22.2)  2/9 (22.2)  2/9 (22.2) | 5/11 (45.5)  4/11 (26.4)  1/11 (9.1)  1/11 (9.1)  0 | 0.210 |
| SOFA  - media (SD)  - mediana (IQR) | 2.2 (1.4)  2 (1-3.50) | 0.82 (1.0)  1 (0-1) | **0.017** |
| SOFA >1 (%) | 6/8 (75) | 2/11 (18.2) | **0.024** |
| Hemogasanalysis, mean (SD)  - pH  - pO2    - pCO2  - Lactates  - SpO2  - P/F RATIO  - HCO3^-^ | 7.45 (0.03)  85.50 (11.33) (median [IQR]:  86 [74-95.75])  27.50 (7.019  2.06 (1.31)  97.33 (1.86)  380.50 (107.78) (median [IQR]:  409.50 [312.25-456.25])  20.70 (2.05) | 7.42 (0.5)  109.14 (60.25) (median [IQR]:  88 [73-119])  36.14 (5.76)  1.17 (0.75)  97.69 (1.62)  339.57 (96.26) (median [IQR]:  348 [240-419])  23.27 (5.10) | 0.280  0.367  **0.033**  0.903  0.721  0.484  0.441 |
| **Laboratory data** | | | |
| Lymphocytes, x10^3^/μL, mean (SD) | 1.31 (0.81) | 1.39 (0.69) | 0.818 |
| PLT, x10^3^/μL, mean (SD) | 284700 (112410) | 248400 (126866) | 0.507 |
| LDH, UI/L, mean (SD) | 447.89 (340.54) | 475.22 (480.10) | 0.891 |
| Albumin, g/dL, mean (SD) | 7.58 (10.0) | 3.87 (0.59) | 0.385 |
| Myoglobin, ng/ml, mean (SD) | 294 (283.95) | 209.0 (295.08) | 0.673 |
| CRP, mg/dL, mean (SD) | 6.59 (15.74) | 4.08 (5.05) | 0.693 |
| D-dimer, μg/L, mean (SD) | 3115.40 (1719.73) | 2801.43 (1432.30) | 0.737 |
| Fibrinogen, μg/L, mean (SD) | 481.0 (91.35) | 491.50 (98.64) | 0.808 |
| Fibrinogen >555 μg/L (%) | 4/10 (40.0) | 5 (50.0) | 1.0 |
| INR, mean (SD) | 1.07 (0.09) | 1.09 (0.21) | 0.803 |
| aPTT, mean (SD) | 0.89 (0.18) | 0.90 (1.13) | 0.901 |
| ATIII, %, mean (SD) | (n=2)  76.55 (6.29) | 0 | - |
| FVIII at baseline, %, mean (SD) | 196.18 (132.84) | - | - |
| vWF:Ag at baseline, %, mean (SD) | 275.39 (184.97) | - | - |
| vWF:RCo at baseline, %, mean (SD) | 219.69 (204.93) | - | - |
| FXIII at baseline, %, mean (SD) | 80.50 (34.60) | - | - |
|  |  |  |  |

Values are presented as mean (±SD), median (interquartile range [IQR]), or number (%) as appropriate.

Student’s t test or Mann-Whitney U test and χ2 or Fisher exact test were used as appropriate.

Abbreviations: ACE-I/ACEIIR-I, angiotensin-converting enzyme - I inhibitors / angiotensin-converting enzyme - II receptor inhibitors; Anti-PF-4, anti-platelet factor 4; aPTT, activated partial thromboplastin time; CE, contrast enhancement; CRP, C-reactive protein; DAPT, double antiplatelet therapy; DOAC, direct oral anticoagulant; HU, Hounsfield unit; ICA, internal carotid artery; IgG, Immunoglobulin G; IQR, interquartile range; INR, international normalized ratio; IV, intravenous; LDH, lactate dehydrogenase; LVO, large vessel occlusion; MCA, middle cerebral artery; MOF, multiorgan failure; mRS, modified Rankin Scale; NIHSS, National Institutes of Health Stroke Scale; P/F ratio, PO2 / FIO2 ratio; SOFA, Sequential Organ Failure Assessment; PLT, platelet; SP, spike protein; SD, standard deviation; SVO, small vessel occlusion; TIA, transient ischemic attack; TICI, thrombolysis in cerebral infarction; TOAST, Trial of Org 10172 in Acute Stroke Treatment; vWFAg, von Willebrand factor antigen; vWFRCo, von Willebrand factor ristocetin cofactor.

**Table S3. Laboratory data at admission in the overall study patient population and by COVID-19**

|  | **All patients**  **(n=39)** | **AIS with**  **COVID-19**  **(n=22)** | **AIS without**  **COVID-19**  **(n=17)** | **p** |
| --- | --- | --- | --- | --- |
| Lymphocytes, x10^3^/μL | 1.54 (0.92) | 1.35 (0.74) | 2.30 (1.25) | **0.035** |
| PLT, x10^3^/μL | 243472 (98691) | 266550 (118136) | 214625 (58746) | 0.118 |
| LDH, UI/L | 355.69 (324.54) | 461.56 (404.03) | 219.57 (47.18) | **0.034** |
| CRP, mg/dL | 5.49 (12.0) | 5.49 (12.0) | - | - |
| D-dimer, μg/L | 2540.78 (1611.53) | 2932.25 (1490.16) | 1757.83 (1685.59) | 0.150 |
| Fibrinogen, μg/L | 452.88 (106.07) | 486.25 (92.69) | 405.21 (108.83) | **0.026** |
| Fibrinogen >555 μg/L (%) | 12/34 (35.3) | 9/20 (45.0) | 3/14 (21.4) | 0.275 |
| INR | 1.09 (0.25) | 1.08 (0.16) | 1.12 (0.34) | 0.625 |
| aPTT | 0.93 (0.18) | 0.89 (0.16) | 0.97 (1.91) | 0.156 |
| FVIII, % | 136.02 (108.66) | 196.18 (132.84) | 87.89 (52.79) | **0.031** |
| vWF:Ag, % | 197.53 (151.62) | 275.39 (184.97) | 128.32 (67.75) | **0.041** |
| vWF:RCo, % | 168.13 (153.17) | 219.69 (204.93) | 126.89 (86.03) | 0.211 |
| FXIII, % | 84.98 (31.61) | 80.50 (34.60) | 88.56 (30.39) | 0.606 |

Values are means (SD) and n (%).

Abbreviations: aPTT, activated partial thromboplastin time; CRP, C-reactive protein; INR, international normalized ratio;

LDH, lactate dehydrogenase; PCT, plateletcrit; PLT, platelet; vWF:Ag, von Willebrand factor antigen; vWF:RCo, von Willebrand factor ristocetin cofactor.

Demographics and clinical characteristics of the AIS patients with COVID-19 undergoing serum SP measurements and by serum positivity to SP are reported herein in the Supplementary Table 3.

Of note, despite both SP-positive and SP-negative had a similar stroke severity at admission as measured by the NIHSS (15 vs 17), no neurological improvement at 24 hours was observed in the SP-positive patients compared to those without SP detectable in the serum (15.50 vs 5). Despite that, the two SP-positive patients had a lower mean infarct volume (10.85 cm^3^ vs 89.13 cm^3^) and a lower COVID-19 severity as measured by the SOFA score (mean, 0.5 vs. 2.7 in the SP-negative patients; SOFA >1: 0 vs 6/7 [85.7%]). However, the numbers are too small for drawing definite conclusions.

**Table S4. Demographics and clinical characteristics of the AIS patients with COVID-19 undergoing serum SP measurements and by serum positivity to SP**

|  | **All patients**  **(n=10)** | **SP positive**  **(n=2)** | **SP negative**  **(n=8)** | **p** |
| --- | --- | --- | --- | --- |
| **Demographics and pre-stroke clinical characteristics** | | | | |
| Age, mean (SD) | 72.8 (17.6) | 76.50 (27.58) | 71.9 (16.8) | 0.760 |
| Sex (females) (%) | 3 (30) | 1 (50) | 2 (25) | 1.0 |
| Pre-mRS (%)  - 0  - 1  - 2  - 3  - 4  - 5 | 6 (60)  1 (10)  2 (20)  0  1 (10)  0 | 1 (50)  0  0  0  1 (50)  0 | 5 (62.5)  1 (12.5)  2 (25)  0  0  0 | 0.188 |
| Hypertension (%) | 7 (70) | 1 (50) | 6 (75) | 1.0 |
| Diabetes mellitus (%) | 5 (50) | 1 (50) | 4 (50) | 1.0 |
| Smoking (current?) (%) | 0 | 0 | 0 | **-** |
| Dyslipidemia (%) | 5 (50) | 0 | 5 (62.5) | 0.444 |
| Ischemic cardiomyopathy (%) | 3 (30) | 0 | 3 (37.5) | 1.0 |
| Atrial fibrillation (%) | 3 (30) | 1 (50) | 2 (25) | 1.0 |
| Previous stroke/TIA (%) | 1 (10) | 0 | 1 (12.5) | 1.0 |
| Anemia (%) | 1 (10) | 0 | 1 (12.5) | 1.0 |
| Hypothyroidism (%) | 0 | 0 | 0 | - |
| Carotid stenosis >50% (%) | 2 (20) | 0 | 2 (25) | 1.0 |
| Valvulopathy (%) | 0 | 0 | 0 | - |
| Neoplasm (%) | 2 (20) | 0 | 2 (25) | 1.0 |
| Antiplatelets (%) | 5 (50) | 1 (50) | 4 (50) | 1.0 |
| Anticoagulants (%) | 2 (20) | 0 | 2 (25) | 1,0 |
| Beta-blockers (%) | 5 (50) | 1 (50) | 4 (50) | 1.0 |
| ACE-I/ACEIIR-I (%) | 6 (60) | 1 (50) | 5 (62.5) | 1.0 |
| Diuretics (%) | 2 (20) | 0 | 2 (25) | 1.0 |
| Calcium antagonists (%) | 1 (10) | 1 (50) | 0 | 0.200 |
| Lipid lowering medications (%) | 4 (40) | 0 | 4 (50) | 0.467 |
| Blood glucose lowering medications (%) | 3 (30) | 1 (50) | 2 (25) | 1.0 |
| **Stroke clinical and radiological characteristics** | | | | |
| Stroke on awakening / unknown time of onset | 6 (60) | 2 (100) | 4 (50) | 0.467 |
| Infarct location (%)  - Right  - Left  - Bilateral  - Subtentorial | 4 (40)  6 (60)  0  0 | 1 (50)  1 (50)  0  0 | 3 (37.5)  5 (62.5)  0  0 | 1.0 |
| Occlusion site (%)  - Top of ICA  - Tandem occlusion  - MCA-M1  - MCA-M2  - MCA-M3-M4  - Posterior circulation  - No occlusion | 1 (10)  0  4 (40)  3 (30)  0  0  2 (20) | 0  0  1 (50)  1 (50)  0  0  0 | 1 (12.5)  0  3 (37.5)  2 (25)  0  0  2 (25) | 0.766 |
| NIHSS at baseline, median (IQR) | 17 (4.75-23) | 15.50 (8-n/a) | 17 (4.25-22) | 0.694 |
| NIHSS at 24 h, median (IQR) | 8 (4-19) | 15.50 (8-n/a) | 5 (2.50-15.50) | 0.245 |
| IV thrombolysis (%) | 1 (10) | 0 | 1 (12.5) | 1.0 |
| Mechanical thrombectomy (%) | 4 (40) | 1 (50) | 3 (27.5) | 1.0 |
| Time from stroke onset to IVT (min)  - mean (SD)  - median (IQR) | n=1  215  - | - | n=1  215  - | - |
| Time from stroke onset to groin puncture (min)  - mean (SD)  - median (IQR) | n=3  555 (120.1)  581 (424-n/a) | - | N=3  555 (120.1)  581 (424-n/a) | **-** |
| Time from onset to recanalization (min) |  |  |  |  |
| Hemorrhagic transformation (%) | 3 (30) | 1 (50) | 2 (25) | 1.0 |
| Collateral circulation (Menon score) (%)  - good1(17-20)  - moderate (16-11)  - poor (10-0) | 0/2  2/2 (100)  0/2 | - | 0/2  2/2 (100)  0/2 | - |
| Collateral circulation (Menon score) (%)  - good/moderate  - poor (10-0) | 2/2 (100)  0/2 | - | 2/2 (100)  0/2 | - |
| Collateral circulation (Menon score), mean (SD) | 12.5 (2.1) | - | 12.5 (2.1) | - |
| TICI  - 2a  - 2b  - 3 | 1/4 (25)  0  3/4 (75) | 0  0  1/1 (100) | 1/3 (33.3)  0  2/3 (66.7) | 1.0 |
| Infarct volume, cm^3^  - mean (SD)  - median (IQR) | 73.47 (108.25)  25.95 (7.80-105.35) | 10.85 (3.61)  - | 89.13 (116.89  41.50 (9-202.45) | 0.296 |
| Secondary prevention therapy (%)  - Aspirin  - Clopidogrel  - DAPT  - Warfarin  - DOAC  - Enoxaparin | 5/6 (83.3)  1/6 (16.7)  0  0  0  0 | 1/1 (100)  0  0  0  0  0 | 4/5 (80)  1/5 (20)  0  0  0  0 | 1.0 |
| TOAST (%)  - LVO  - Cardioembolic  - SVO  - Cryptogenic  - Other determined causes | 2 (20)  3 (30)  0  5 (50)  0 | 0  1 (50)  0  1 (50)  0 | 2 (25)  2 (25)  0  4 (50)  0 | 0.659 |
| **Characteristics of COVID-19** | | | | |
| Time from COVID-19 diagnosis and stroke  (days)  - mean (SD)  - median (IQR) | 6.5 (8.7)  4 (0-12.25) | 0  - | 9.8 (9.1)  8.50 (2-18.75) | 0.227 |
| Anti-SP antibodies (IgG), positive (≥4.81) (%) | 1/4 (25) | 0/1 | 1/3 (33.3) | 1.0 |
| Anti-SP antibodies (IgG), BAU/ml  (test range 4.81-2080)  - mean (SD)  - median (IQR) | (n=4)  390.20 (759.91)  13 (4.80-1152.80) | (n=1)  4.80  - | (n=3)  518.67 (875.88)  21.20 (1530.0-21.20) | 0.662 |
| SP, pg/ml, mean (SD) | 0.38 (0.80) | 0.38 (0.80) | 0 | **<0.001** |
| Anti-PF4 positive (%) | 0 | 0 | 0 | - |
| ACE2 positive (%) | 0 | 0 | 0 | - |
| Chest CT (%) | 9 (90) | 2 (100) | 7 (87.5) | 1.0 |
| Pneumonia (%)  - <20% (mild)  - 20-50% (moderate)  - >50% (severe)  - Bilateral pleural effusion | 8 (80)  1/9 (11.1)  3/9 (33.3)  3/9 (33.3)  2/9 (22.2) | 1 (50) | 7 (87.5)  0/7  2/7 (28.6)  3/7 (42.9)  2/7 (28.6) | 0.378 |
| Sepsis (%) | 0 | 0 | 0 | - |
| MOF (%) | 0 | 0 | 0 | - |
| SOFA (%)  - 0  - 1  - 2  - 3  - 4 | 1/9 (11.1)  2/9 (22.2)  2/9 (22.2)  2/9 (22.2)  2/9 (22.2) | 1 (50)  1 (50)  0  0  0 | 0/7  1/7 (14.3)  2/7 (28.6)  2/7 (28.6)  2/7 (28.6) | 0.191 |
| SOFA  - media (SD)  - mediana (IQR) | 1.5 (1.4)  1 (0-2.75) | 0.5 (0.7)  - | 2.7 (1.1)  3 (2-4) | **0.036** |
| SOFA >1 (%) | 6/8 (75) | 0 | 6/7 (85.7) | 0.250 |
| Hemogasanalysis, mean (SD)  - pH  - pO2    - pCO2  - Lactates  - SpO2  - P/F RATIO  - HCO3^-^ | 7.45 (0.03)  85.50 (11.33) (median [IQR]:  86 [74-95.75])  27.50 (7.019  2.06 (1.31)  97.33 (1.86)  380.50 (107.78) (median [IQR]:  409.50 [312.25-456.25])  20.70 (2.05) | n=1  7.42  75.0  30.0  0.70  96.0  357.0  - | 7.45 (0.03)  87.60 (11.28) (median [IQR]:  88 [77.50-97.50])  27.0 (7.71)  2.40 (1.23)  97.60 (1.95)  385.20 (119.81) (median [IQR]:  419 [289-464.50])  20.70 (2.05) | 0.422  0.366  0.740  0.304  0.495  0.840 |
| **Laboratory data** | | | | |
| Lymphocytes, x10^3^/μL | 1.31 (0.81) | 0.84 (0.02) | 1.43 (0.88) | 0.384 |
| PLT, x10^3^/μL | 284700 (112410) | 202500 (26163) | 305250 (117198) | 0.271 |
| LDH, UI/L | 447.89 (340.54) | 199.50 (3.54) | 518.86 (358.03) | 0.269 |
| Albumin, g/dL | 7.58 (10.0) | - | 7.58 (10.0) |  |
| Myoglobin, ng/ml | 294 (283.95) | 74 (57.98) | 440.67 (460.96) | 0.366 |
| CRP, mg/dL | 6.59 (15.74) | 0.39 (0.13) | 8.37 (17.72) | 0.563 |
| D-dimer, μg/L | 3115.40 (1719.73) | - | 3115.40 (1719.73) |  |
| Fibrinogen, μg/L | 481.0 (91.35) | 401.0 (118.79) | 501.0 (80.17) | 0.179 |
| Fibrinogen >555 μg/L (%) |  |  |  |  |
| INR | 1.07 (0.09) | 1.09 (0.08) | 1.06 (0.10) | 0.781 |
| aPTT | 0.89 (0.18) | 0.80 (0.23) | 0.91 (0.18) | 0.467 |
| ATIII, % | (n=2)  76.55 (6.29) | - | (n=2)  76.55 (6.29) | - |
| FVIII at baseline, %, mean (SD) | 196.18 (132.84) | (n=1)  118.0 | 207.34 (139.36) | 0.571 |
| vWFAg at baseline, %, mean (SD) | 275.39 (184.97) | (n=1)  128.0 | 296.44 (189.15) | 0.437 |
| vWFRCo at baseline, %, mean (SD) | 219.69 (204.93) | (n=1)  41.0 | 245.21 (207.16) | 0.392 |
| FXIII at baseline, %, mean (SD) | 80.50 (34.60) | (n=1)  71.0 | 81.86 (37.14) | 0.794 |

Values are presented as mean (±SD), median (interquartile range [IQR]), or number (%) as appropriate.

Student’s t test or Mann-Whitney U test and χ2 or Fisher exact test were used as appropriate.

Abbreviations: ACE-I/ACEIIR-I, angiotensin-converting enzyme - I inhibitors / angiotensin-converting enzyme - II receptor inhibitors; Anti-PF-4, anti-platelet factor 4; aPTT, activated partial thromboplastin time; CE, contrast enhancement; CRP, C-reactive protein; CT, computerized tomography; DAPT, double antiplatelet therapy; DOAC, direct oral anticoagulant; HU, Hounsfield unit; ICA, internal carotid artery; IgG, Immunoglobulin G; IQR, interquartile range; INR, international normalized ratio; IV, intravenous; LDH, lactate dehydrogenase; LVO, large vessel occlusion; MCA, middle cerebral artery; MOF, multiorgan failure; MRI, magnetic resonance imaging; mRS, modified Rankin Scale; NIHSS, National Institutes of Health Stroke Scale; P/F ratio, PO2 / FIO2 ratio; SOFA, Sequential Organ Failure Assessment; PLT, platelet; SP, spike protein; SD, standard deviation; SVO, small vessel occlusion; TIA, transient ischemic attack; TICI, thrombolysis in cerebral infarction; TOAST, Trial of Org 10172 in Acute Stroke Treatment; vWFAg, von Willebrand factor antigen; vWFRCo, von Willebrand factor ristocetin cofactor.
