# Supplementary material for: SARS-CoV-2 infection predicts larger infarct volume in patients with acute ischemic stroke
Source: Front Cardiovasc Med. 2023 Jan 10;9:1097229. doi: 10.3389/fcvm.2022.1097229 (PMC9871539; doi:10.3389/fcvm.2022.1097229)
Supplement: Supplementary file 2 [file Image_1.PDF]

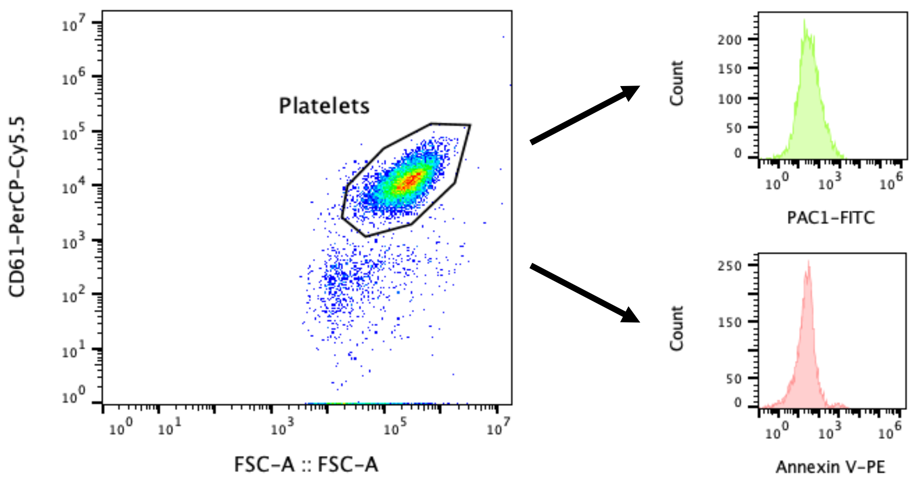

Figure S1. Flow cytometry gating strategy used to evaluate the activation of the platelet-specific integrin αIIbβ3 (by measuring PAC1-FITC binding), and the phosphatidylserine (PS) exposure (by measuring annexin V-PE binding) on the surface of of washed healthy platelets incubated with sera from acute ischemic stroke (AIS) patients, with or without COVID-19, or of healthy controls (HC). Platelets were gated on a forward scatter FSC/CD61-PerCP-Cy5.5 plot (on the left) to select only CD61+ cells, and then the median fluorescence intensity (MFI) were evaluated for PAC1-FITC (green) and Annexin V-PE (red).
