# Supplementary material for: SARS-CoV-2 infection predicts larger infarct volume in patients with acute ischemic stroke
Source: Front Cardiovasc Med. 2023 Jan 10;9:1097229. doi: 10.3389/fcvm.2022.1097229 (PMC9871539; doi:10.3389/fcvm.2022.1097229)
Supplement: Supplementary file 3 [file Image_2.PDF]

A.

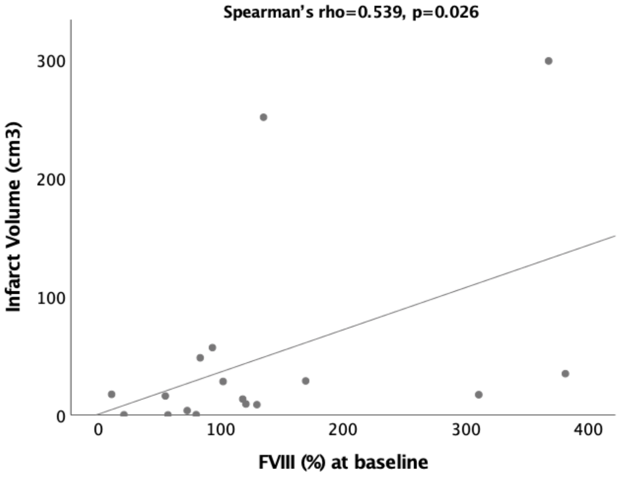

B.

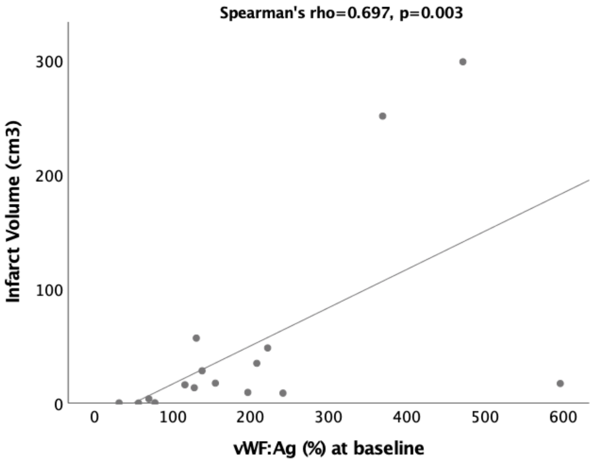

Figure S2. Correlation analysis by using the Spearman's rho test between infarct volume and baseline levels of FVIII (n=17) (A) and vWF:Ag (n=16) (B) in the overall study population
