# Supplementary material for: SARS-CoV-2 infection predicts larger infarct volume in patients with acute ischemic stroke
Source: Front Cardiovasc Med. 2023 Jan 10;9:1097229. doi: 10.3389/fcvm.2022.1097229 (PMC9871539; doi:10.3389/fcvm.2022.1097229)
Supplement: Supplementary file 4 [file Image_3.PDF]

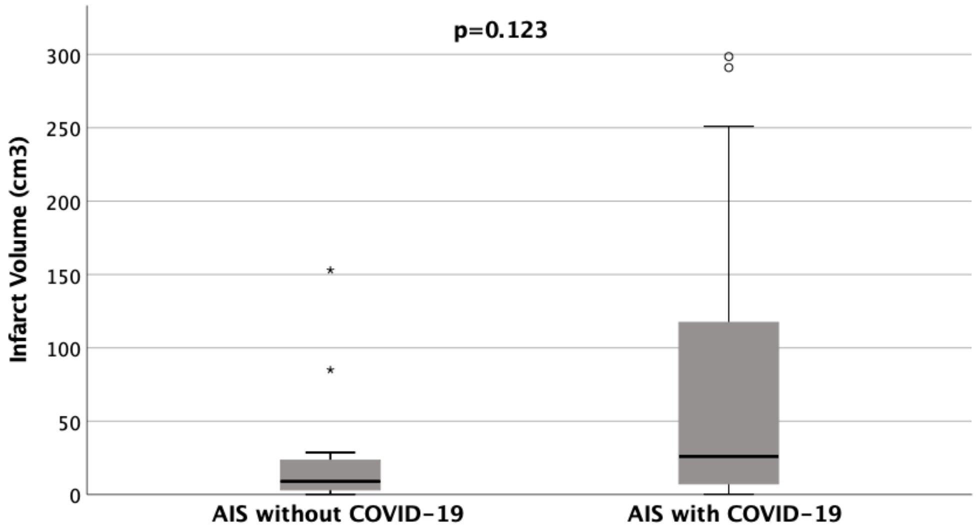

Figure S3. Differences in median (IQR) values of infarct volume between acute ischemic stroke (AIS) patients without COVID-19 (n=16) and those with COVID-19 (n=20) (Student t test, p=0.063; Mann-Whitney U test, p=0.123) (the asterisks ** and the small circles °° indicates the outliers (i.e., patients who presented extreme values of infarct volume) for the without-COVID-19 and with-COVID-19 groups, respectively.
